# Correction: Luminescence properties of Bi^3+^/Sm^3+^ co-doped K_3_Gd_5_(PO_4_)_6_ phosphors for self-referencing optical thermometry

**DOI:** 10.1039/d4ra90130d

**Published:** 2024-11-06

**Authors:** Jing Wang, Mingjun Song, Junpeng Xue, Shala Bi, Hyo Jin Seo

**Affiliations:** a School of Chemistry and Chemical Engineering, Weifang University Weifang Shandong China smj521209@126.com; b School of Science, Jiangsu University of Science and Technology Zhenjiang China; c Department of Physics, Pukyong National University Busan 608-737 South Korea

## Abstract

Correction for ‘Luminescence properties of Bi^3+^/Sm^3+^ co-doped K_3_Gd_5_(PO_4_)_6_ phosphors for self-referencing optical thermometry’ by Jing Wang *et al.*, *RSC Adv.*, 2024, **14**, 31398–31408. https://doi.org/10.1039/D4RA06205A.

The authors would like to clarify the affiliation list of the published article to correctly show where the work was conducted. In the original article, the address at the time of publication of one author (Shala Bi) was listed as an affiliation. However, the work was conducted at the Department of Physics, Pukyong National University. The corrected list of affiliations at the time of publication of the original article is presented above.

The Royal Society of Chemistry apologises for these errors and any consequent inconvenience to authors and readers.

